# Rhabdomyolysis Following Ad26.COV2.S COVID-19 Vaccination

**DOI:** 10.3390/vaccines9090956

**Published:** 2021-08-27

**Authors:** Georg Gelbenegger, Filippo Cacioppo, Christa Firbas, Bernd Jilma

**Affiliations:** 1 Department of Clinical Pharmacology, Medical University of Vienna, 1090 Vienna, Austria; georg.gelbenegger@meduniwien.ac.at (G.G.); christa.firbas@meduniwien.ac.at (C.F.); 2 Department of Emergency Medicine, Medical University of Vienna, 1090 Vienna, Austria; filippo.cacioppo@meduniwien.ac.at

**Keywords:** COVID-19, vaccine, Ad26.COV2.S, rhabdomyolysis, myoglobinuria

## Abstract

We report the case of a 19-year-old male who complained of myalgia, muscle weakness, and darkened urine two days after receiving his Ad26.COV2.S (Johnson & Johnson, New Brunswick, New Jersey, United States) COVID-19 vaccination. Blood examination revealed an increased creatine kinase (CK) level, and his urinary dipstick tested positive for blood, indicative of acute rhabdomyolysis. Serum creatinine levels were normal. Rhabdomyolysis due to strenuous physical activity was ruled out and further diagnostics excluded an autoimmune cause. Under repeated treatment with intravenous fluid resuscitation (outpatient treatment), his symptoms resolved and peak CK levels of 44,180 U/L returned to almost normal levels within two weeks. Rhabdomyolysis is a rare, potentially fatal vaccine-induced reaction. Further research is needed to better understand the underlying pathomechanism and to investigate whether subcutaneous injection of vaccines may be able to prevent rhabdomyolysis.

## 1. Introduction

The recent outbreak of the coronavirus disease 2019 (COVID-19), caused by the severe acute respiratory syndrome coronavirus 2 (SARS-CoV-2), has presented a major challenge for health care systems worldwide. With the long-awaited emergence of vaccines against SARS-CoV-2, vaccination side-effects are once again in the spotlight. While common reactions include injection site pain, fever, malaise, and headache, other more dangerous side-effects, such as vaccine-induced immune thrombotic thrombocytopenia, were reported [1].

Ad26.COV2.S is a recombinant, replication-incompetent adenovirus serotype 26 vector encoding a full-length membrane-bound SARS-CoV-2 spike protein in a prefusion-stabilized conformation [2,3]. A single dose of Ad26.COV2.S protected against symptomatic COVID-19 infection and was effective against severe-critical disease [4]. Safety of Ad26.COV2.S appeared to be similar to that in other phase 3 trials of COVID-19 vaccines [4] with further monitoring of adverse events taking place in a post-marketing setting.

Rhabdomyolysis is a serious condition characterized by skeletal muscle injury which can lead to acute kidney failure. We report the case of a 19-year-old previously healthy man, who developed severe rhabdomyolysis two days after receiving the Ad26.COV2.S COVID-19 vaccine.

## 2. Case Report

A 19-year-old male presented to the Department of Clinical Pharmacology of the Medical University of Vienna for participation in a clinical trial (unrelated to the COVID-19 vaccination). During the screening visit, which was performed two weeks prior to vaccination, he was in good health, had a normal physical examination and unremarkable laboratory findings. He had no relevant past medical history (no past COVID-19 infection), was not taking any medication, and had no known allergies. Prior reactions to vaccinations included increased body temperature, which was usually self-limiting. He was an avid swimmer, training regularly once a week.

Two days prior to his first clinical trial visit, he went to the gym to perform light exercise, which was not more strenuous than usual. In the evening of the same day, he received his COVID-19 vaccination (Ad26.COV2.S, Johnson & Johnson, New Brunswick, New Jersey, United States), injected into his left upper arm.

On the following day (day 1 after vaccination), he noticed light muscle soreness (numeric rating scale of pain, NRS 3). Around noon, a mild increase in body temperature (37.5 °C) occurred, which subsided after intake of acetaminophen. In the afternoon, pain and immobility worsened, especially in his elbows, shoulders, back, and wrists (NRS 7). As the pain hindered the subject’s sleep, he took another dose of acetaminophen during the night.

On day 2 after vaccination, he reported to our department for participation in a clinical trial. Before planned administration of the study medication, he complained of muscle weakness and whole-body pain (NRS 10), which was aggravated at rest and alleviated when in motion. His vital signs were normal, and his electrocardiogram showed no signs of ischemia or myocarditis. His urine was reddish-brown, testing strongly positive for blood in the dipstick test. His urine sediment was weakly positive for red blood cells and his urinary drug test was negative.

Laboratory results revealed a creatine kinase (CK) level of 15,638 U/L, serum creatinine of 1.06 mg/dL, a lactate dehydrogenase (LDH) level of 428 U/L and elevated liver enzymes (aspartate transaminase (AST) 340 U/L, alanine transaminase (ALT) 70 U/L), C-reactive protein 1.61 mg/dL. The high-sensitivity cardiac troponin T was below measurable limits. Antinuclear antibodies, ENA subsets (SSA(Ro), SSB(La), SCL-70, SM, u1RNP, RNP70, Jo-1, centromere B), anti-Jo-1, PM/Scl-100, anti-PL-7, anti-PL-12, anti-Mi-2, anti-Ku(p70/80), and anti-SRP antibodies were negative. Table 1 shows the clinical and laboratory findings over time. Due to the abnormal clinical and laboratory findings and accompanying safety concerns, study medication was not infused, and he was excluded from the trial.

**Figure 1:**
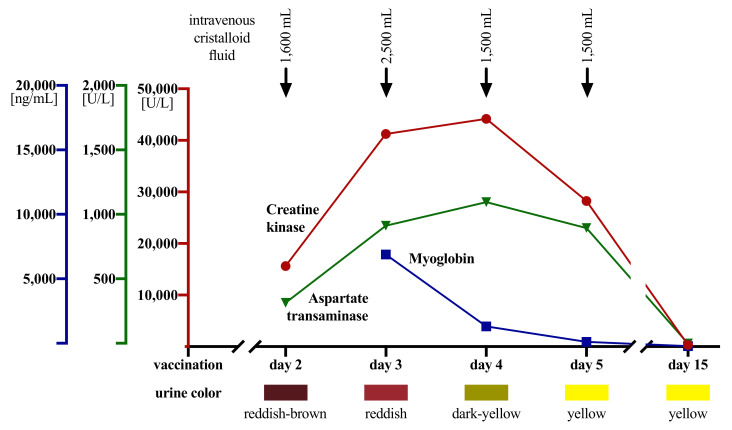
Time course of laboratory findings, urinalyses, and interventions.

Intravenous volume resuscitation was started to avoid myoglobin precipitation and tubule obstruction causing acute kidney failure (Figure 1). In addition, he was advised to increase his daily fluid intake to 4 L per day and monitor his urine output and weight. Intravenous acetaminophen was given once for pain relief. Furthermore, he was ordered to rest and abstain from any physical activity. As the patient insisted on going home after infusion therapy, an ambulatory management plan was agreed upon.

He returned to our department the next day (day 3 after vaccination) for a follow-up. Pain was improved (NRS 6–7), and overall, he felt better. However, his follow-up laboratory results showed a further increase in CK level to 41,260 U/L, rising AST and ALT levels, a myoglobin level of 7146 U/L and a stable serum creatinine of 0.93 mg/dL. His urine output was normal and intravenous fluid resuscitation was continued.

CK levels continued to rise during the next follow-up visit (day 4 after vaccination), reaching a peak level of 44,180 U/L. Clinically, the patient continued to improve (NRS 5), his urinary dipstick test returned to normal, and he kept receiving intravenous fluids.

Finally, on day 5 after vaccination, CK levels decreased for the first time, dropping to 28,260 U/L. As his myalgia continued to improve (NRS 2), the patient insisted on going on vacation the same day, which precluded any further timely follow-up visits.

He returned to our department on day 15 after vaccination in good health (NRS 0) with his CK level having returned to near normal (Table 1).

## 3. Discussion

Rhabdomyolysis is defined as destruction of striped (skeletal) muscle cells with subsequent release of its contents, most notably electrolytes, myoglobin, creatine kinase, lactate dehydrogenase, alanine aminotransferase, and aspartate aminotransferase into the circulation [5]. The pathomechanisms of rhabdomyolysis are direct sarcolemmic injury (in trauma: additional muscle cell injury due to ischemia-reperfusion and neutrophil-caused inflammation) or depletion of adenosine triphosphate within the myocyte, causing uncontrolled calcium influx and leading to collapse of the myofibrillar network and subsequent myocyte death [6,7,8,9].

The most serious complication of rhabdomyolysis is acute kidney failure, which can occur in 13 to 50% of cases [10,11,12]. The underlying mechanism of acute kidney failure caused by rhabdomyolysis is unclear, but intrarenal vasoconstriction, tubule injury (mostly in the proximal tubule), and tubular obstruction (mostly in the distal tubule) appear to be causative. Myoglobin is freely filtered in the glomeruli and is then taken up into tubule epithelial cells via endocytosis and metabolized. At high concentrations, myoglobin precipitates in the renal tubules, which is promoted by acidic urine [13]. When serum myoglobin levels exceed 100 mg/dL, myoglobin is overtly visible in the urine [14].

Our patient was a previously healthy 19-year-old male, who developed severe muscle pain one day after Ad26.COV2.S vaccination. He presented to our department with the classic triad of rhabdomyolysis: myalgia, muscle weakness, and myoglobinuria. Intravenous fluid resuscitation and oral hydration likely averted the development of acute kidney failure, in spite of significantly increased serum creatine kinase and myoglobin levels. Typically of rhabdomyolysis, our patient tested strongly positive for blood in the urine dipstick test (inability of the dipstick test to differentiate between hemoglobin and myoglobin) but without a significant RBC count. Under ambulatory treatment, his clinical symptoms improved, and his laboratory results returned to baseline at a follow-up visit 15 days later.

Cases of rhabdomyolysis associated with COVID-19 vaccinations have been reported before (Table 2) [15,16,17]. To our knowledge, this is the first published case of rhabdomyolysis following COVID-19 vaccination with the Ad26.COV2.S vaccine, although similar incidences have been captured in the vaccine adverse event reporting system (VAERS) (Table 3). In total, rhabdomyolysis was complicated by acute kidney injury in four cases with one fatal outcome, highlighting the potentially serious course of disease. However, it remains unclear whether the development of acute kidney failure in these cases was due to the Ad26.COV2.S vaccine or was multicausal.

The occurrence of rhabdomyolysis following vaccination is not only limited to COVID-19 vaccines but also extends to vaccines against other viruses, e.g., against the influenza virus [18]. The mechanism behind vaccine-induced rhabdomyolysis is largely unknown. In general, vaccinations (more specifically adjuvants) may trigger the release of myotoxic cytokines such as tumor necrosis factor alpha, which has been shown to induce skeletal muscle breakdown [19].

Another potential mechanism may be the generation of cross-reacting antibodies targeting skeletal muscle cells leading to myositis. We did not test our patient for myositis-autoantibodies. The differential diagnosis of myositis was unlikely, as symptom onset was deemed too early for timely antibody formation. Furthermore, myositis-antibodies were negative and his eventual response to aggressive fluid resuscitation without the use of glucocorticoids or non-steroidal antirheumatics rendered an autoimmune cause unlikely.

COVID-19 itself has also been associated with rhabdomyolysis [20,21]; however, the mechanism of muscle damage caused by viral infections has not been established. A suggested common pathomechanism of rhabdomyolysis in disease and vaccination can only be speculated.

Further questions remain regarding whether rhabdomyolysis occurs only after intramuscular injection of vaccines and is associated with the combination of muscle trauma and immune response to the vaccine, and whether it could be prevented by subcutaneous injections.

## 4. Conclusions

Physicians should be aware of the possibility of vaccine-induced rhabdomyolysis as a potentially serious side-effect in patients complaining of myalgia following vaccination, particularly in times of mass vaccinations. Patients may be advised to monitor the color of their urine for the early detection of rhabdomyolysis. Finally, our case report suggests the need for aggressive fluid resuscitation and close monitoring for the prevention of acute kidney failure in patients with rhabdomyolysis.

## Figures and Tables

**Table 1:** Timeline of clinical and laboratory findings.

	Screening Visit	Day 2 (After Vaccination)	Day 3	Day 4	Day 5	Day 15
**Lab parameter**						
CK (<190 U/L)	163	15,638	41,260	44,180	28,260	347
GOT (<50 U/L)	15	340	938	1121	920	24
GPT (<50 U/L)	21	70	237	309	354	51
LDH (<250 U/L)	163	428	784	875	608	211
Myoglobin (23–72 ng/mL)	ND	ND	7146	1576	371	50
Creatinine (0.7–1.2 mg/dL)	0.96	1.06	0.93	0.82	0.92	0.97
**Urinary dipstick**						
color		reddish-brown	reddish	dark yellow	yellow	yellow
pH (4.5–7.4)		5	6	6	NA	7
blood (hemoglobin *)		positive	positive	negative	negative	negative
**Pain (NRS)**	0	10	6–7	5	2	0

* The urinary dipstick test is unable to differentiate between hemoglobin and myoglobin. (ND = not done, NA = not available).

**Table 2:** Summary of published cases of rhabdomyolysis associated with COVID-19 vaccinations.

Case Report	Vaccine	Age, Sex	Time to Symptom Onset	Peak CK Level	Acute Kidney Failure	Outcome	Note
Tan et al., 2021	ChAdOx1 nCoV-19 (AstraZeneca)	27, m	5 h	250,000 U/L	no	recovered	known CPT2 deficiency
Mack et al., 2021	mRNA-1273 (Moderna)	80, m	2 days	6546 U/L	no	recovered	-
Nassar et al., 2021	BNT161b2 mRNA (Pfizer/BioNTech)	21, m	1 day	>22,000 U/L	no	recovered	-

**Table 3:** Summary rhabdomyolysis cases following vaccination with Ad26.COV2.S, as captured in the vaccine adverse event reporting system (VAERS) (https://wonder.cdc.gov/vaers.html), last accessed on 24th August 2021.

VAERS ID	Age, Sex	Time to Symptom Onset	Peak CK Level [U/L]	Acute Kidney Failure	Outcome	Note
1177103-1	47, m	1 day	1700	yes	recovered	sepsis with unknown source of infection
1211823-1	37, f	6 days	>32,000	no	recovered	-
1223359-1	30, m	5 days	95,694	no	recovered	notable recent moderate exercise an over-ingestion of gabapentin; under methadone, quetiapine, lithium, gabapentin, nicotine vape
1225589-1	22, f	7 days	127,768	no	recovered	HIIT training, not more strenuous than usual, NKDA
1227789-1	unknown, f	< 1 day	unknown	no	recovered	no rhabdomyolysis
1282506-1	86, m	4 weeks	unknown	no	recovered	ischemic stroke, likely traumatic rhabdomyolysis
1286545-1	81, f	21 days	unknown	no	recovered	evidence of rhabdomyolysis/traumatic rhabdomyolysis?
1293926-1	71, f	28 days	unknown	no	recovered with permanent disability	ischemic stroke, likely traumatic rhabdomyolysis; concomitant simvastatin treatment
1316930-1	21, f	3 days	204,900	no	recovered	-
1336145-1	37, f	17 days	744,000	yes	unknown	CT scan suggested myositis in her masseter muscles, transfer to ICU, intubated, RRT
1345707-1	unknown	unknown	>500,000	no	unknown	-
1371923-1	49, m	8 days	unknown	no	recovered	-
1388426-1	61, m	19 days	unknown	no	unknown	de novo Guillain-Barré syndrome, rhabdomyolysis due to statin intake
1391630-1	86, m	3 days	unknown	yes	fatal	sepsis, bilateral pneumonia, acute kidney injury; PMHx tumor in lungs, AFIB, hyperlipidemia
1423267-1	72, f	28 days	unknown	unknown	recovered with permanent disability	sepsis, UTI, pneumonia, SVT, NSTEMI; under atorvastatin
1430442-1	unknown	unknown	~750,000	yes	recovered with permanent disability	INT, tracheostomy, 19 days in ICU; myoglobin > 5000
1441330-1	61, f	19 days	>15,000	no	recovered	current COVID-19 infection, statin-induced rhabdomyolysis
1450792-1	57, f	4 days	unknown	unknown	unknown	hospitalized, unknown days in hospital
1512116-1	41, f	6 days	16,000	no	recovered	crossfit as possible confounding error
1519231-1	30, m	8 days	unknown	unknown	unknown	under citalopram

VAERS was last accessed on 24th August 2021. The only search term used was “rhabdomyolysis”. The search yielded 20 results. Not all reported events were directly vaccine-induced. A search for “rhabdomyolysis” comprising all COVID-19 vaccines yielded 232 results (24th August 2021). Units of measurements were not always reported (CT = computed tomography, NKDA = no known drug allergies, RRT = renal replacement therapy, AFIB = atrial fibrillation, INT = intubation, ICU = intensive care unit, UTI = urinary tract infection, NSTEMI = non-ST-elevation myocardial infarction, SVT = supraventricular tachycardia).

## Data Availability

The authors declare that the data supporting the findings of this study are available within the paper. The data that support the findings of this study are available from the corresponding author upon reasonable request. No identifying information can be released.
